# Thickness-Dependence Electrical Characterization of the One-Dimensional van der Waals TaSe_3_ Crystal

**DOI:** 10.3390/ma12152462

**Published:** 2019-08-02

**Authors:** Bum Jun Kim, Byung Joo Jeong, Seungbae Oh, Sudong Chae, Kyung Hwan Choi, Tuqeer Nasir, Sang Hoon Lee, Hyung Kyu Lim, Ik Jun Choi, Min-Ki Hong, Hak Ki Yu, Jae-Hyun Lee, Jae-Young Choi

**Affiliations:** 1 SKKU Advanced Institute of Nanotechnology (SAINT), Sungkyunkwan University, Suwon 16419, Korea; 2 School of Advanced Materials Science & Engineering, Sungkyunkwan University, Suwon 16419, Korea; 3 Department of Materials Science and Engineering, Department of Energy Systems Research, Ajou University, Suwon 16499, Korea

**Keywords:** TaSe_3_, mechanical exfoliation, work function, van der Waals crystal, scanning Kelvin probing microscopy

## Abstract

Needle-like single crystalline wires of TaSe_3_ were massively synthesized using the chemical vapor transport method. Since the wedged-shaped single TaSe_3_ molecular chains were stacked along the b-axis by weak van der Waals interactions, a few layers of TaSe_3_ flakes could be easily isolated using a typical mechanical exfoliation method. The exfoliated TaSe_3_ flakes had an anisotropic planar structure, and the number of layers could be controlled by a repeated peeling process until a monolayer of TaSe_3_ nanoribbon was obtained. Through atomic force and scanning Kelvin probe microscope analyses, it was found that the variation in the work function with the thickness of the TaSe_3_ flakes was due to the interlayer screening effect. We believe that our results will not only help to add a novel quasi-1D block for nanoelectronics devices based on 2D van der Waals heterostructures, but also provide crucial information for designing proper contacts in device architecture.

## 1. Introduction

The demand for novel device architecture and materials has been increasing tremendously due to the physical limitations of the current Si-based semiconductor technology, which arise as the size of a single transistor decreases to nanometer size [1,2,3,4]. Even though the issues arising from high-density integration in electronic manufacturing have been partly solved using a three-dimensional (3D) gate structure, more fundamental solutions should be proposed to meet the requirements for the new era of artificial intelligence technology, for which quicker data processing with a small amount of energy is required [5,6,7,8,9]. Among the alternatives, two-dimensional (2D) layered materials with a single atomic thickness have been regarded since the past decade as strong candidates for overcoming the Si-based technology, thanks to their superior mechanical, physical, and chemical properties compared to conventional 3D bulk materials [2,4,10,11,12,13]. In particular, by using the 2D van der Waals (vdWs) heterostructure, in which the stacking sequence and angle can be controlled at the atomic level, semiconductor devices with a thickness of a few nanometers can be manufactured [4,12,13]. Additionally, a new quantum physical phenomenon can be observed [13]. However, in order to achieve high integration for mass production and the desired device structure, micro-patterning processes are inevitable. Unfortunately, the method of selectively etching a specific layer in vdWs heterostructure is still challenging, and it can lead to a drastic degradation of the electrical characteristics because of extensive dangling bonds at the edge sites [11]. To address the aforementioned problems, research has been conducted on the synthesis and application of chain-based layered materials, which can be used directly as quasi-1D conducting channels in a 2D vdWs heterostructure without an additional patterning process [14]. Several research groups have successfully demonstrated that Mo_6_S_9_–_x_I_x_ [15], Nb_2_Se_9_ [16,17,18], V_2_Se_9_ [19,20,21,22], and TaSe_3_ [14,23,24,25,26,27,28,29,30,31,32] consisting of multiple single molecular chains linked by weak vdWs interaction, can be exfoliated and used in electronic devices, optoelectronic devices, and energy storage devices. Recently, Balandin et al. researched the trigonal prismatic TaSe_3_ material, on which superconducting properties were observed decades ago [14]. The authors found that this material had a high current-carrying capacity, and had the potential of being employed as an interconnector in electronic devices [18]. Shur et al. further proved that the quasi-1D nanowires of TaSe_3_ had lower levels of normalized noise spectral density, and had a potential for downscaled local interconnect applications [24,27,31]. However, despite the nano-effects of layered materials observed in the few-layered regions, previous studies of TaSe_3_ only considered materials with a diameter of 20 nm or greater. In particular, for use as a component of the conducting channels of the 2D vdWs heterostructure, it is ultimately necessary to assess the electrical characteristics at sizes ranging from monolayer to multi-layers. In this study, we synthesized bundles of TaSe_3_ crystal by a typical chemical vapor transport (CVT) method, and cleaved them to a thickness of a few layers through a simple mechanical exfoliation approach. Exfoliated TaSe_3_ flakes have an anisotropic 2D structure with a flat surface, which shows that as-grown TaSe_3_ crystals consist of innumerable single TaSe_3_ molecular chains bonded by the weak vdWs interaction along the b-axis. Topological characteristics of TaSe_3_ were investigated by atomic force microscopy (AFM), and the calculated thickness-dependent work functions were analyzed by a scanning Kelvin probe microscope (SKPM).

## 2. Materials and Methods

### 2.1. Synthesis

**Figure 1:**
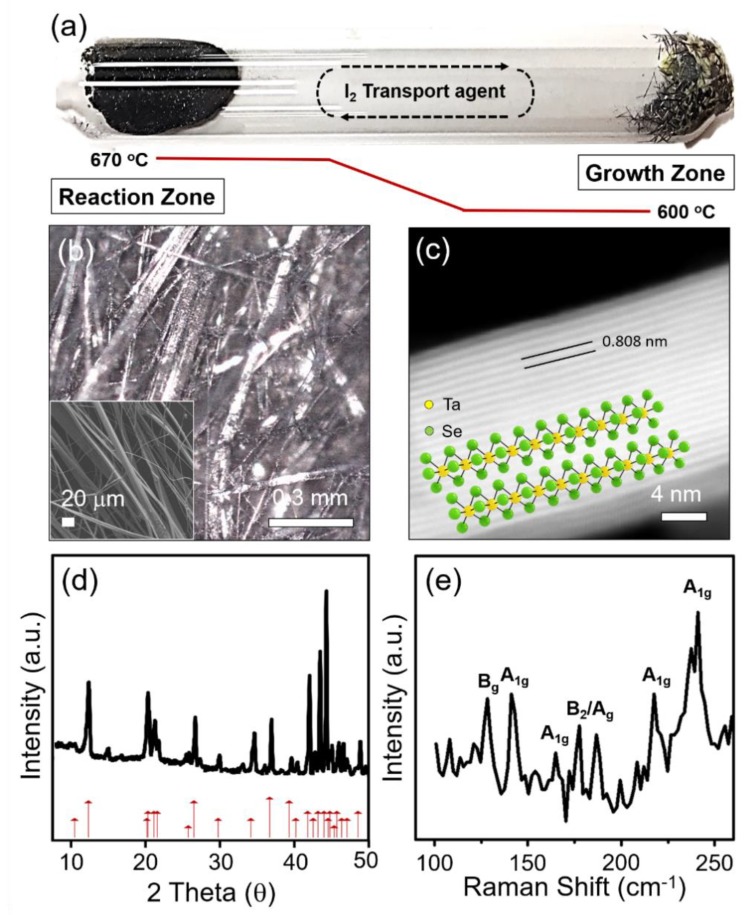
(**a**) Photograph of the sealed evacuated quartz ampoule after the chemical vapor transport (CVT) process for TaSe_3_ crystal growth. (**b**) OM image of entangled needle-like TaSe_3_ single crystals. The inset shows a high-magnification FE-SEM image of TaSe_3_ crystals. (**c**) Scanning transmission electron microscopy (STEM) image of TaSe_3_ flakes obtained by chemical exfoliation. The inset is an illustration of the crystal structure of the TaSe_3_. (**d**) XRD pattern of the bulk TaSe_3_. (**e**) Micro-Raman spectra of the bulk TaSe_3_ on a 300 nm SiO_2_/Si substrate.

The single-crystalline TaSe_3_ was prepared by the CVT method (as described in Figure 1a). For the growth of TaSe_3_, stoichiometric amounts of tantalum powder (0.42 g, Alfa Aesar, 99.97%, Haverhill, MA, USA) and selenium powder (0.58 g, Alfa Aesar, 99.999%, Haverhill, MA, USA), with iodine (10 mg, Sigma Aldrich, 99.999%, Saint Louis, MO, USA) as a transport agent, were placed in a quartz tube (18 × 1 cm) that was subsequently evacuated and sealed. After that, the tube was placed in a home-made two-zone tube furnace. The reaction zone was slowly heated to 670 °C, and maintained at constant temperature for 10 days to allow for the compound synthesis. Similarly, the temperature of the growing zone was maintained at 600 °C (heating rate: 200 °C/h). Finally, the furnace was switched off, and cooled down to room temperature (cooling rate: 50 °C/h).

### 2.2. Mechanical Exfoliation

As-grown bulk TaSe_3_ was placed on a wafer dicing tape (BT150EKL, Nitto, Umeda, Japan) and stuck several times to obtain a thinner-than-bulk material. A 300 nm SiO_2_/Si substrate was cleaned by ultrasonication in acetone, ethanol, and deionized water for 15 min, followed by heating at 100 °C to remove moisture from the surface. The polymer tape was then pressed firmly, and adhered to the 300 nm SiO_2_/Si substrate. After adhesion, the polymer tape was removed from the substrate, and the process was repeated.

### 2.3. Characterization

Powder X-ray diffraction (XRD) (Mac Science, M18XHF22, Tilburg, the Netherlands) was performed using Cu–Kα radiation (λ = 0.154 nm), and a step size of 0.04/s. Optical Microscope (OM) (OLYMPUS, BX51M, Tokyo, Japan) was performed in bright field condition. AFM (Park systems, NX10, Suwon, Korea) was performed in non-contact mode for the topographic analysis. Field emission scanning electron microscopy (FE-SEM) (Hitachi, S4300SE, Tokyo, Japan) and scanning transmission electron microscopy (STEM) (JEOL, JEM-2100F, Tokyo, Japan) were used to evaluate morphology and crystallinity of exfoliated TaSe_3_. For TEM imaging, TaSe_3_ flakes ultrasonicated in an ethanol solution were dispersed onto a Cu grid with a lacy carbon support film, and imaged at an accelerating voltage of 200 kV. Raman spectroscopy (HORIBA, LabRAM HV, Kyoto, Japan) with excitation energy of 1.58 eV (785 nm, 5 mW) was used to characterize the TaSe_3_ flakes on a 300 nm SiO_2_/Si substrate. SKPM (Park systems, NX10, Suwon, Korea) measurements were performed using n-type Si tips coated with Cr–Au (NSC36/Cr–Au, Mikromash Inc., Watsonville, CA, USA) at a resonance frequency of 65 kHz, scan rate of 0.5 Hz, and sample bias of ±1 V. The Cr–Au tip was calibrated by highly ordered pyrolytic graphite ($\varphi_{\mathrm{HOPG}}$ = 4.65 eV) and the calculated work function of the Cr–Au tip was about 4.88 eV.

## 3. Results and Discussion

Shiny and needle-like TaSe_3_ wires are obtained by the CVT method (see Section 2 and Figure 1a,b). As shown in the inset of Figure 1b, individual TaSe_3_ bundles—tens of nanometers in diameter—have weak vdWs forces of attraction, and thus can be separated into multiple strands of chains during sample preparation for FE-SEM. The STEM image of the exfoliated TaSe_3_ flake showed continuous and clean lattice fringes of 0.808 nm gap, corresponding to the (1, 0, −1) plane (Figure 1c). The crystalline structure of the TaSe_3_ was further characterized using XRD and Raman spectroscopy as shown in Figure 1d,e. Strong XRD peaks of as-grown TaSe_3_ wires were consistent with the peaks from the crystallography database of TaSe_3_ (red arrows in Figure 1d, JCPDS-04-007-1143). Raman spectra for a single TaSe_3_ wire were obtained under a laser power of 5 mW to avoid laser-induced rupturing and heating effects. The obtained Raman spectra exhibited several distinct peaks at 100 to 260 cm^−1^, originating from the prismatic chain structure of metal trichalcogenide [14,27,33,34]. The peaks at 141, 164, 217, and 237 cm^−1^ corresponded to the out-of-plane (A_1g_) vibration mode, while those at 177 and 186 cm^−1^ represented vibration symmetry of chain (B_2_) and crystal (A_g_). The relatively strong peak at 128 cm^−1^ represented the shearing (B_g_) vibration of the chains, indicating a strong Ta–Se intrachain bond of TaSe_3_.

**Figure 2:**
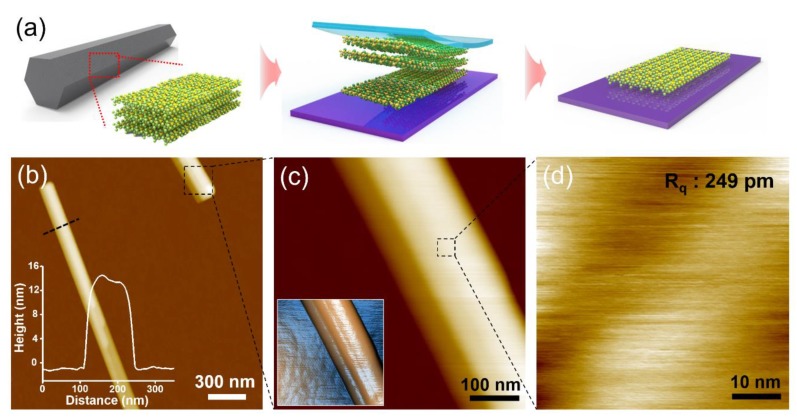
(**a**) Schematic illustration for the mechanical exfoliation of TaSe_3_ flakes from bulk TaSe_3_ crystal using the Scotch tape method and (**b**–**d**) atomic force microscopy (AFM) images of exfoliated TaSe_3_ flakes on 300 nm SiO_2_/Si substrate. Inset of (b) shows line profile of the corresponding TaSe_3_ flake, as marked in (b) and inset of (c) shows 3D AFM image of the exfoliated TaSe_3_ flake.

To verify the layered nature of the bulk TaSe_3_ crystals, the well-known micromechanical cleavage technique was employed (Figure 2a) [2]. Figure 2b–d shows AFM images of the exfoliated TaSe_3_ flakes with different thicknesses transferred on the 300 nm SiO_2_/Si substrate. Like other 2D materials (e.g., graphene and h-BN), the color of the flakes changed from yellowish to bluish as the thickness decreased (see the AFM images in Figure S1) [35,36]. The surface of the exfoliated flake was observed to have a semi-infinite planar structure, indicating that TaSe_3_ is a quasi-1D vdWs material and can be employed as an important component of the 2D vdWs heterostructure. 

**Figure 3:**
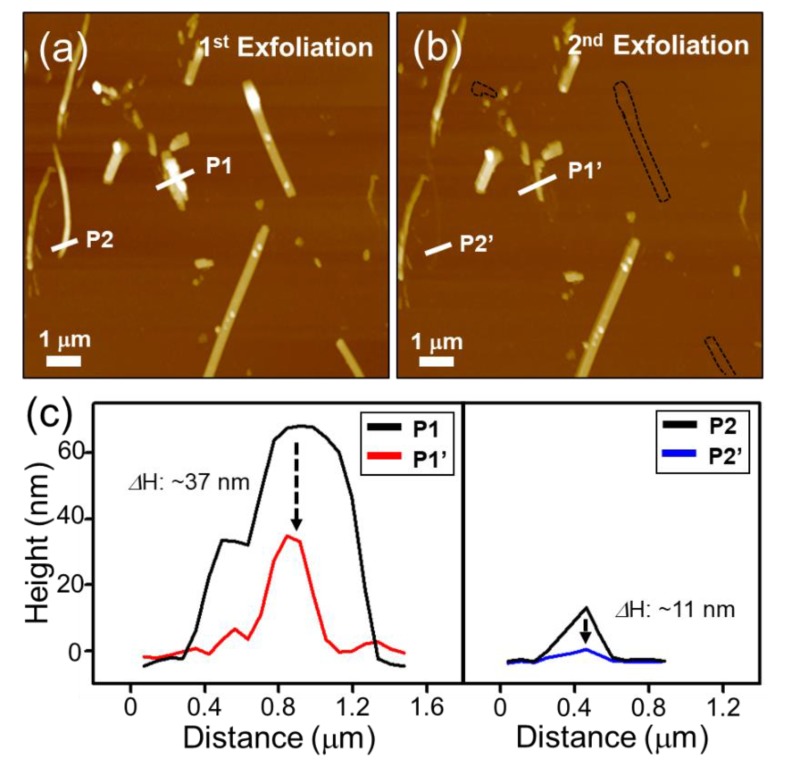
(**a**) AFM image of the first exfoliated TaSe_3_ nanoribbon on a 300 nm SiO_2_/Si substrate, (**b**) AFM image of second exfoliated TaSe_3_ nanoribbon on a 300 nm SiO_2_/Si substrate, and (**c**) line profile graph before and after exfoliation of the TaSe_3_ bundles.

By using a repeated peeling method [16,21], the thickness of the exfoliated TaSe_3_ flakes could be controlled. Figure 3 shows that the thickness of the TaSe_3_ flakes decreased from 70 to 36 nm (P1 to P1’), and from 16 to 5 nm (P2 to P2’), after an additional exfoliation process at the same location using an adhesive tape. Some parts of the TaSe_3_ flakes disappeared, and are marked as a black dotted line in Figure 3b.

**Figure 4:**
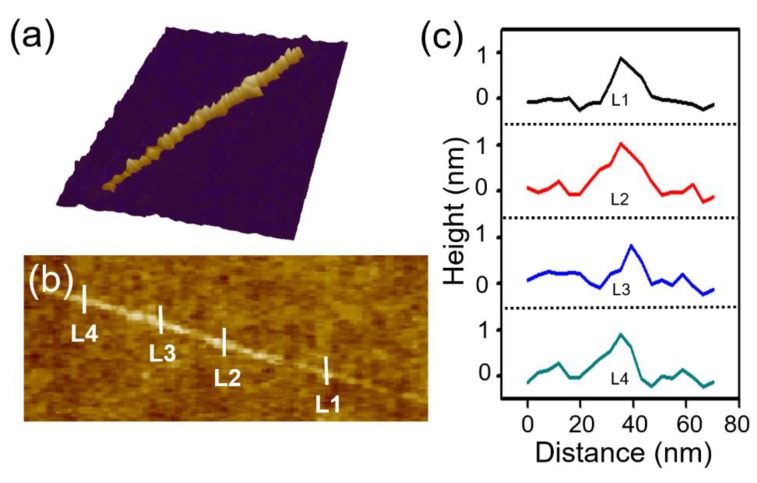
(**a**) 3D image of the monolayer of a quasi-1D TaSe_3_ nanoribbon on a 300 nm SiO_2_/Si substrate after multiple peelings, (**b**) AFM image of an isolated monolayer TaSe_3_ nanoribbon, and (**c**) line-profile graph marked as L1, L2, L3, and L4 in Figure 4b.

Eventually, we obtained a monolayer of TaSe_3_ nanoribbons (TaSe_3_ structure with a single molecule chain thickness of 0.8 nm, and width of approx. 20 nm) on a 300 nm SiO_2_/Si substrate (Figure 4). We expect that the obtained monolayer of TaSe_3_ nanoribbon can be applied to 2D heterostructures as a quasi-1D conduction channel with ideal transport characteristics.

**Figure 5:**
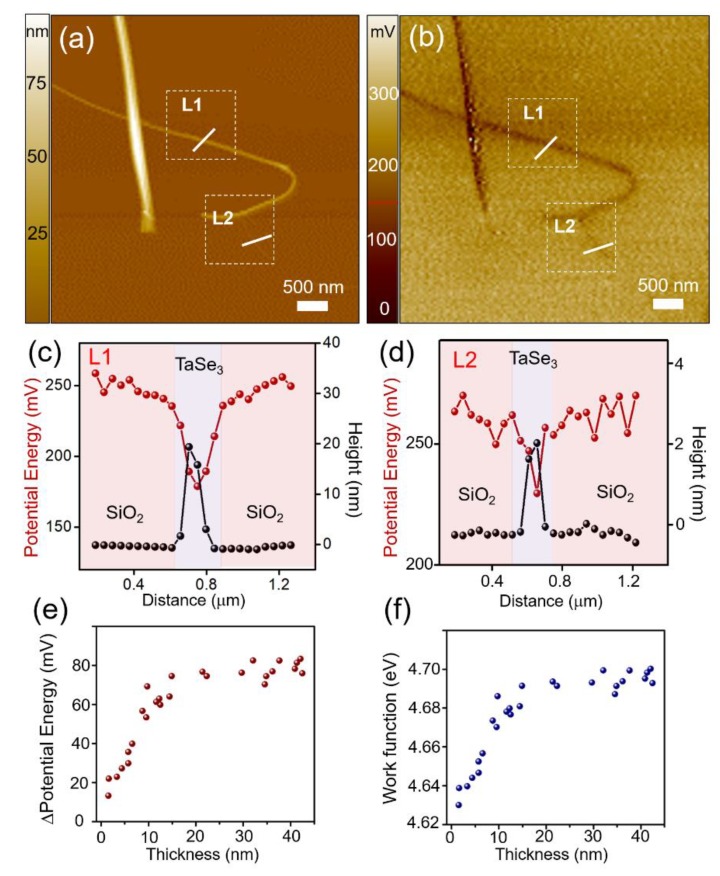
(**a**) AFM height image; (**b**) identical SKPM image of the exfoliated TaSe_3_ flakes on a 300 nm SiO_2_/Si substrate; (**c**,**d**) height/potential energy profiles of the flakes in (a) and (b); and (**e**,**f**) variations in the potential energy difference and work function, respectively, according to the thickness of the TaSe_3_ flakes.

The difference in electrical properties according to the number of layers (i.e. thickness) of the exfoliated TaSe_3_ flakes was evaluated through SKPM analysis. The local surface potential energy difference and the work function were determined by measuring the contact potential difference between the tip and the sample (V_CPD_) [35,37,38]. Since the TaSe_3_ bundle was transferred to a 300 nm SiO_2_/Si substrate, the work function could be calculated using the following equations:

(1) $$V_{CPD}=\frac{1}{e}\left(\varphi_{t}-\varphi_{f}\right)$$

(2) $$∆V_{CPD}=V_{CPD}\left(TaSe_{3}\right)-V_{CPD}\left(substrate\right)$$

(3) $$=\frac{1}{e}\left(\varphi_{t}-\varphi_{f}\right)-\frac{1}{e}\left(\varphi_{t}-\varphi_{s}\right)$$

(4) $$=\frac{1}{e}\left(\varphi_{s}-\varphi_{f}\right)$$

where $\varphi_{t}$, $\varphi_{s}$, and $\varphi_{f}$ represent the work functions of the tip, SiO_2_, and TaSe_3_, respectively. Figure 5a,b show an AFM and SKPM image, respectively, of the same TaSe_3_ flakes on a 300 nm SiO_2_/Si substrate, while Figure 5c,d illustrate how the value of the surface potential energy varied with the TaSe_3_ thickness, as marked in L1 and L2, respectively. The thickness and potential energy difference of L1 were approx. 22 nm (approx. 25 layers) and 70 mV, respectively, and those of L2 were approx. 2 nm (approx. 2 layers) and 25 mV, respectively. From the results of further measurements of 27 samples with different thickness, we are able to confirm that, as the thickness of the TaSe_3_ flake decreased below 24 nm, the surface potential energy difference and work function sharply decreased (Figure 5e,f). The results for the thickness dependence work function variation will be useful for selecting a suitable contact material for future nano-devices that could have a significant impact on performance. These results appear to be caused by the interlayer screening effect, which is commonly observed in other exfoliated layered nanomaterials on substrate. In addition, in comparison with the graphene (~2 nm) and MoS_2_ (~5 nm), the TaSe_3_ had longer screening length (24 nm), indicating that chemical property of the TaSe_3_ might be hydrophilic [35,37,38,39].

## 4. Conclusions

In this study, we successfully demonstrated that needle-like TaSe_3_ crystals can be exfoliated to a chain-based quasi-1D layered structure. The thickness (or number of layers) of TaSe_3_ flakes was controlled by repeated exfoliation and, eventually, a monolayer TaSe_3_ nanoribbon was obtained. Through AFM and SKPM analysis, we verified that the change in the work function depended on the thickness of the TaSe_3_ flakes due to the interlayer screening effect. We anticipate that our results will help in developing and designing next-generation devices based on 2D vdWs heterostructure.

## Supplementary Materials

The following are available online at https://www.mdpi.com/1996-1944/12/15/2462/s1, Figure S1: (a) OM and (b) AFM image of exfoliated TaSe_3_ flakes on 300 nm SiO_2_/Si substrate. (c) Line profile of the corresponding TaSe_3_ flake, as marked in (b).
