# Supplementary material for: Thickness-Dependence Electrical Characterization of the One-Dimensional van der Waals TaSe3 Crystal
Source: Materials (Basel). 2019 Aug 2;12(15):2462. doi: 10.3390/ma12152462 (PMC6695938; doi:10.3390/ma12152462)
Supplement: Supplementary file 1 [file materials-12-02462-s001.pdf]

Supplementary

Thickness-Dependence Electrical Characterization of the One-Dimensional van der Waals $TaSe_3$ Crystal

Bum Jun Kim [1,†], Byung Joo Jeong [2,†], Seungbae Oh [2], Sudong Chae [2], Kyung Hwan Choi [1], Tuqeer Nasir [1], Sang Hoon Lee [2], Hyung Kyu Lim [2], Ik Jun Choi [2], Min-Ki Hong [3], Hak Ki Yu [3], Jae-Hyun Lee [3,*] and Jae-Young Choi [1,2,*]

1 SKKU Advanced Institute of Nanotechnology (SAINT), Sungkyunkwan University, Suwon 16419, Korea
2 School of Advanced Materials Science & Engineering, Sungkyunkwan University, Suwon 16419, Korea
3 Department of Materials Science and Engineering, Department of Energy Systems Research, Ajou University, Suwon 16499, Korea
* Correspondence: jaehyunlee@ajou.ac.kr (J.-H.L.); jy.choi@skku.edu (J.-Y.C.)
† These authors contributed equally to this study

Received: 16 June 2019; Accepted: 30 July 2019; Published: 2 August 2019

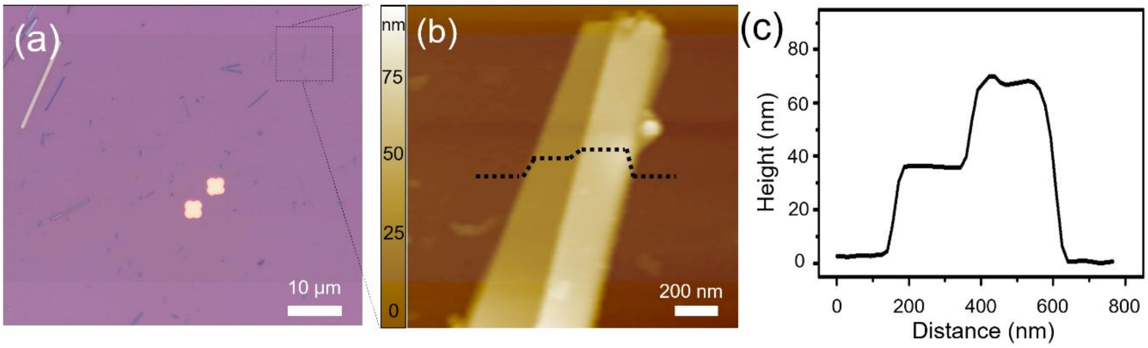

Figure S1. (**a**) OM and (**b**) AFM image of exfoliated TaSe3 flakes on 300 nm SiO_2/Si substrate. (**c**) Line profile of the corresponding $TaSe_3$ flake, as marked in (**b**).

© 2019 by the authors. Licensee MDPI, Basel, Switzerland. This article is an open access article distributed under the terms and conditions of the Creative Commons Attribution (CC BY) license (http://creativecommons.org/licenses/by/4.0/).
